# Understanding the impact of a collective leadership intervention on team working and safety culture in healthcare teams: a realist evaluation protocol

**DOI:** 10.12688/hrbopenres.12860.2

**Published:** 2020-03-26

**Authors:** Aoife De Brun, Lisa Rogers, Marie O'Shea, Eilish McAuliffe

**Affiliations:** 1 School of Nursing, Midwifery and Health Systems, University College Dublin, Dublin, Ireland

**Keywords:** collective leadership, realist evaluation, healthcare, team working, safety culture, intervention, evaluation

## Abstract

There is accumulating evidence for the value of collective and shared approaches to leadership across sectors and settings. However, relatively little research has explored collective leadership in healthcare and thus, there is little understanding of what works for healthcare teams, why, how and to what extent. This study describes the approach that will be adopted to the realist evaluation of a collective leadership intervention with four heterogenous healthcare teams in four different settings. A realist evaluation will be conducted. Realist evaluation is a theory-based approach to evaluation. It enables the use of mixed-methods to explore the research question of interest. Development of an initial programme theory (IPT) constitutes the first phase of the approach. This IPT will be informed by interviews with members of teams identified as working collectively, an examination of extant literature using realist synthesis, and will be refined through consultation with an expert panel. A multiple case study design will be adopted to explore the impact of the intervention, including quantitative scales on teamworking, leadership and safety culture, realist interviews with key informants and observations of teams during intervention sessions. Analysis of data will be guided by the IPT to refine the theory and context-mechanism-outcome configurations. Findings from the cases will be compared to identify patterns or demi-regularities and to explore if the intervention operates differently in different contexts. This analysis and synthesis of findings across the teams will inform the development of a middle range theory that will not only add to our understanding of how collective leadership influences teamwork and patient safety, but also provide guidance for future collective leadership interventions.  Favourable ethical opinion has been received from the University College Dublin Ethics Committee. Results will be disseminated via publication in peer-review journals, national and international conferences and to stakeholders/interest groups.

## Introduction

Leadership has been described as the most influential factor in shaping organisational culture to enable health service improvement ^1^. While effective leadership can positively impact healthcare settings, recent patient safety reports have implicated failures in leadership, or a lack of leadership, as key factors in safety shortcomings ^2, 3^. There have been calls for a shift from the traditional, heroic single-leader focus, towards a more inclusive, shared approach to leadership ^4– 8^, where leadership is not located in one individual, but instead is a property of the team and something that can be shared to fit with task demands. This collective approach has been defined as a dynamic team phenomenon, where leadership roles are distributed ^9^ and shared among the team, and individuals adopt leadership roles where they have the expertise and motivation to do so ^10^. Although collective and shared approaches to leadership have been found to enhance team effectiveness and team performance outcomes ^9– 11^, there remains a dearth of research on collective leadership in healthcare settings. While there is some emerging evidence for the effectiveness of collective leadership interventions in healthcare ^12^, we do not have insight into how these interventions work, why they work, and the contexts in which they may be more or less effective.

A recent systematic review highlights the scarcity of research on collective leadership interventions in healthcare ^12^. The research that has been published indicates the positive impact of collective leadership interventions on staff engagement, quality improvement, teamworking and patient satisfaction ^12^. The studies retrieved in the review were largely focused on effectiveness of interventions rather than on the mechanisms through which they operate. Thus, we have some understanding of what works, but there is a lack of insight into how and why these interventions work, and how context may influence their impact. This is an important gap that needs to be addressed, as an enhanced understanding of the mechanisms that trigger outcomes in specific contexts can enable researchers and practitioners to develop strategies to support the successful implementation of evidence-based interventions. This realist evaluation not only seeks to determine whether this collective leadership intervention is effective or not but also to delve deeper to document and analyse how and why an intervention may prove successful (or not).

The co-designed collective leadership intervention to be evaluated is best described as a complex intervention as it involves multiple stakeholders, intervention components are interrelated and/or interdependent, involve complex tasks and will likely have multiple and variable outcomes ^13^. The intervention has been co-designed by healthcare staff, patient representatives and researchers during six half-day workshops and one full-day workshop over a seven-month period. This co-design process and the resulting intervention has been described in a previous paper ^14^ and has already been evaluated. Thus, this realist evaluation will focus on the implementation of the intervention. Briefly, the intervention comprises a minimum of eight components: six one-hour ‘core’ components related to collective leadership for team performance and collective leadership for safety culture and at least two further intervention components may be selected that are targeted towards specific team types, team needs and/or team goals. There is a total suite of 13 targeted interventions that teams can select to complete; however, the six foundational components are compulsory and a core first phase for all teams. All component interventions at each phase take one hour to complete and will be implemented by the team in their regular work environment. A summary of the full suite of intervention components in Table 1.

**Table 1.** Summary of Co-Lead programme components.

CORE COMPONENTS (to be completed by all teams)	1.	Team Values, Vision and Mission
	2.	Team Goal Setting
	3.	Role Clarity
	4.	Collective Leadership for Safety Skills
	5.	Risk and Safety Management at the Team Level
	6.	Monitoring and Communicating Safety at Team level
TARGETED COMPONENTS (to be selected by teams based on their needs/priorities)	7.	Effective Team Meetings
	8.	Removing Frustrations/Blockers
	9.	Building Trust
	10.	Structured Interdisciplinary Rounds
	11.	Challenging Unsafe Behaviours
	12.	Communication at Safety Critical Moments
	13.	Talking about Safety (PlayDecide Patient Safety game)
	14.	Safety Pause Huddles
	15.	High Reliability at the Team Level
	16.	Developing a Positive Work Environment
	17.	Emotional Support in Teams
	18.	Enhancing Person-Centred Care
	19.	Sustaining Improvements

*Note*: All components available open access at http://www.ucd.ie/collectiveleadership/resourcehub/toolkit/

There is considerable debate in the literature as to whether traditional, positivist methods are appropriate for the study of complex interventions, where researchers have little or no control over the research setting. These approaches have been branded as both an oversimplification of the research environment and of the intervention, as linear approaches to implementation and causality cannot be assumed ^15– 17^. What is needed is an approach that acknowledges this complexity, recognises that research is being conducted in a complex, open system, and considers the significant role of context in implementation and evaluation ^15, 16, 18^. According to Greenhalgh and Papoutsi ^16^, “the study of complexity in health services and systems requires new standards of research quality, namely (for example) rich theorising, generative learning, and pragmatic adaptation to changing contexts” (p. 1). Furthermore, such research offers richer insights to policymakers, researchers and practitioners and is arguably a better means to achieve successful knowledge translation due to the enhanced detail and nuance such an evaluation would confer. For these reasons, we will adopt a realist approach to evaluating this novel intervention.

## Realist evaluation

Pawson and Tilley ^18^ were among the early proponents of realist evaluation and described it as a theory-driven approach to evaluation grounded in scientific realism. They argued that there was a need to understand more than intervention effectiveness and asserted that in order for evaluations to be useful, researchers needed to explore ‘what works for whom, in what context, to what extent, how and why’ ^18^. Thus, realist evaluation is a logic of inquiry that penetrates below the surface level inputs and outputs of an intervention and interrogates the inner mechanisms (M), that is, the implicit reactions and reasonings ^18, 19^, that trigger or inhibit certain intervention outcomes (O) in specific contexts (C) of implementation. The approach is focused on exploring the relationships or configurations between the contexts, mechanisms and outcomes, and therefore the method seeks to explore these configurations through generation and testing of ‘CMOCs’ (i.e., C + M = O).

The aim of realist evaluation is to develop an initial programme theory (IPT) which represents an explanatory framework of theories underlying a programme or intervention. This IPT will then be refined through multiple method data collection. The CMOCs generated in the early IPT guide this process and more CMOCs may be added or removed through an iterative process of data collection and analysis. The ultimate aim of realist evaluation is to produce a middle range theory (MRT). It is recognised that this MRT is not a grand theory, but rather a theory that can be further refined and tested through future realist evaluations. This MRT is not intended as an exhaustive account of all possible or all observed CMOCs, but rather represents a generalisable, transferable account of what works for whom, how and in what context ^18^.

Realist evaluation as an approach is still developing and there is no standardised approach to conducting a realist evaluation ^20^. It is a method-neutral approach wherein methods should be selected to align with the research question and objectives, as well as ensure appropriate testing and refinement of the IPT. In order to evaluate the impact of a complex intervention in complex, open systems, multifaceted analytical strategies are often required, encompassing varied data collection methods.

## Aims, objectives and research questions

The aim of this study is to systematically investigate key features of contexts, mechanisms and outcomes and their interactions by building and testing an explanatory theory to interrogate how collective leadership interventions can be effectively implemented to lead to desired outcomes (improved team working, safety culture and practice of collective leadership).

### Research questions

- Does the intervention effectively enhance team working and patient safety culture and what works for whom, how, to what extent and under what circumstances?

### Objectives

- To describe and provide insight into the contextual conditions evident in the sites of study and understand how these contexts may be linked to mechanisms and outcomes.
- To describe how the intervention is working (or not) in specific implementation sites (mechanisms) and explore its impact (outcomes) based on the perceptions of staff engaging with the intervention and observation field notes.
- To identify demi-regularities across implementation sites and develop context-mechanism-outcome configurations within cases (refined programme theory) and across all cases.

### Protocol

***Context of the research.*** This research will be conducted within one of the seven hospital groups in Ireland. This group, the Ireland East Hospital Group, represents the largest and most complex of the hospital groups, consisting of 11 hospitals (six voluntary and five statutory) in the east of Ireland ranging from small speciality hospitals to large, academic teaching hospitals. Together the hospital group employs over 10,000 staff and serves a population of 1.1 million people in the region. The teams selected to take part in this research represent four different team types spread over multiple sites within the hospital group. Teams were selected and invited to take part based on an attempt to include diverse team types and in line with the priorities of the hospital group. Participation was on a voluntary basis by team agreement following a presentation from the research team that outlined the study aims and design.

## Realist evaluation cycle

**Figure 1.**
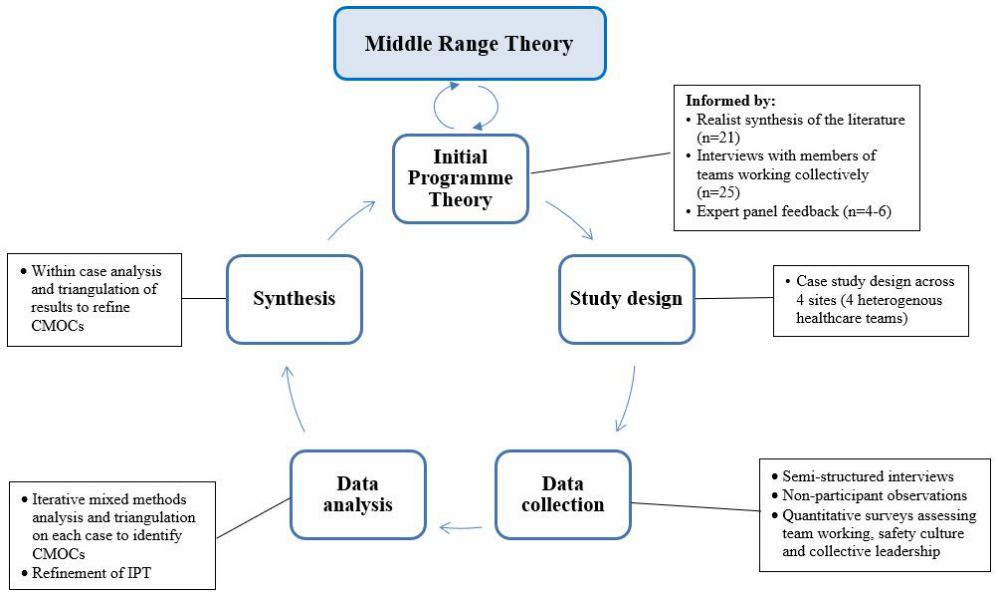
Realist evaluation approach.

The evaluation of the study intervention will be guided by the realist evaluation cycle approach described by Pawson and Tilley ^18^ ( Figure 1). This cycle is explained in the following section alongside an explanation of the methods and approaches that will be adopted for the planned realist evaluation. Figure 1 summarises these steps, data collection methods, procedures and anticipated outcomes for this study.

We started the process with a number of candidate theories about how we expected the intervention to operate to influence outcome. Two of these broad theories which underpinned the rationale and development of the intervention are include below in Box 1.

**Box 1.**

*Candidate theory 1:* In contexts where teams come together to engage in on-site training (as compared to off-site), the training is more likely to be perceived as relevant and valuable to delivery of care and thus the intervention is more likely to be implemented and the team is more likely to work collectively during implementation, with this collective approach subsequently embedding as the team’s leadership style.

*Candidate theory 2:* In contexts where care is delivered by multidisciplinary healthcare teams, introduction of the intervention will enhance staff engagement and develop a more collective mindset. This will result in more positive work environment, staff satisfaction, improved teamworking and improved safety culture.

### Step 1. Formulating the initial programme theory (IPT)

To develop the IPT, the research team will draw on multiple data sources to inform the hypotheses and theory underpinning the intervention. This will involve: (i) a realist synthesis of the literature on collective leadership interventions in healthcare; (ii) interviews with members of teams who have been identified as successfully working collectively in the healthcare system; and (iii) feedback/input on IPT from expert panel members. Further information on the methods of each of these phases within Step 1 are described below.

***Realist synthesis.*** A realist synthesis of the literature will be conducted on papers previously retrieved during a systematic review of interventions to develop collective leadership in healthcare settings (see paper by De Brun *et al*. for full search strategy) ^12^. Our systematic review was conducted with the purpose of identifying approaches to developing collective leadership. Studies were eligible if they reported on the development, evaluation and/or implementation of training or interventions to foster collectivistic approaches to leadership. With this strong focus on development and implementation, as compared to a review that is focused on effectiveness only, we synthesised many papers that were rich in detail and provided a good starting point in the theory-building process. A total of 21 papers will be evaluated and assessed for rigour and ability to add to the developing programme theory; thus, not necessarily all papers will have sufficient information to contribute to theory building. To identify contextual conditions that enabled or inhibited mechanisms for collective leadership in practice, information will be gathered specific to the type of intervention, the setting in which the intervention occurred, contextual data on factors that enabled or inhibited effectiveness, mechanisms that were enacted, and outcomes of the intervention. This information will be collated via a data extraction template that will be designed for this purpose and that will be applied uniformly across all papers. Once this is complete, context-mechanism-outcome configurations (CMOCs) will be extrapolated from each paper where there is sufficient information included to do so. After this, demi-regularities, or common patterns across studies, will be explored.

***Interviews with individuals on teams working collectively.*** In order to test and refine these evolving theories, additional data will be collected to inform the programme theory prior to undertaking the evaluation. With the assistance of senior leaders in the health system, Experts in the health system will help the research team identify up to four healthcare teams that are currently working collectively and effectively in the healthcare system. These teams have had no involvement in the co-design and have not received the intervention. Using Kozlowski and Ilgen’s ^21^ (2006) taxonomy, a team will be defined for the purposes of this research as (a) two or more individuals who (b) socially interact, (c) possess one or more common goals, (d) are brought together to perform organisationally relevant tasks, (e) exhibit interdependence with respect to workflow, goals and outcomes, (f) have different roles and responsibilities and (g) are together embedded in an encompassing organisational system with boundaries and linkages to the broader system context and task environment. We will discuss the concept and definitions of collective leadership (presented earlier) with senior leaders in the system and ask them to identify potential teams for inclusion based on these definitions. Individuals from identified teams will be invited to take part in a one-on-one interview with a researcher to explore their experiences of working within the team, why they felt the team was working collectively, the advantages and disadvantages of working this way and the impact on team working and safety culture. The interview guide has been informed by the intervention components identified in the systematic review of interventions to develop collective leadership in healthcare settings ^12^. The same data extraction template used to extrapolate information from the literature will be used to extrapolate CMOCs from the interview data. The findings will be used to further refine the IPT.

***Expert panel discussions.*** To further advance and refine the IPT, a panel will be convened to gather the views of programme designers and experts in the fields of collective leadership, team working, and patient safety. The expert panel (n=4-6) will assist the research team in refining and finalising the IPT, and, if necessary, prioritising CMOCs for testing. The IPT will also be presented to a panel of researchers experienced in realist evaluation methodology to confirm plausibility and for refinement before it is finalised for testing. This programme theory will be tested and further developed through the subsequent stages of the realist evaluation process.

### Step 2. Study design

Consistent with best practice in realist evaluation, the study design adopted for the next phase is informed by the IPT and the most appropriate methods to test the CMOCs ^18^. In consultation with the senior management in the hospital group of study, teams will be invited to be included in the study to support a range of team types and geographic dispersion across the region. While not possible to include all types of healthcare team in this limited pilot study, we intend to include an acute care team, an integrated care team (across hospital and community), a management team and a surgical team. These four teams will be the only teams to test the intervention and will enable the team to test the intervention prior to wider testing across the hospital group. Given that we are evaluating the implementation of the intervention in four different settings and four heterogenous teams over a one-year period, a multiple case study approach ^22^ is the most appropriate design to understand potential differences regarding how the intervention may trigger different mechanisms and/or lead to different outcomes under different contextual conditions. In this study, one case is defined as one team that is implementing the collective leadership intervention. Four case studies will effectively facilitate cross-case comparisons to detect common CMOCs across cases (i.e., the common thread underlying the intervention in each context). Realist evaluation is iterative and flexible by nature, enabling researchers to respond to findings as they emerge and if necessary redirect researchers to additional or alternative data collection methods.

### Step 3. Data collection

A key advantage of realist evaluation is that it is a method-neutral approach and the most appropriate methods are determined by the research questions, the subject of study and by the IPT. In this study, we will employ a mixed-methods approach to data collection which will include individual semi-structured and realist interviews ^23^, quantitative surveys and field notes gathered during non-participant observations of the teams’ intervention sessions. We will also use a context mapping framework at each site to understand the factors that may impact on implementation and intervention success.

We will invite members from each of the teams to take part in one-on-one semi-structured realist interviews ^23^ with a member of the research team. The interview questions will explore their experience of the intervention, what worked and did not work and why, and how they believe the intervention may have led to certain outcomes. We will seek to recruit up to 10–12 interviewees per research site, or until data saturation is reached. Data collection will be staggered post-intervention, aligning to the time periods when teams complete the intervention. This will enable flexibility in the use of Manzano’s theory-driven approach to interviewing and allow the research team to iteratively refine and test CMOCs throughout data collection.

Members of the research team will also engage in non-participant observation of intervention sessions and intervention planning sessions. A bespoke observation template informed by the Consolidated Framework for Implementation Research (CFIR) ^24^ will be used in each context along with researchers’ field notes of the sessions.

Finally, quantitative data sources will also be utilised. Where teams have selected goals for improvement during the intervention period that reflect data already being gathered by/for teams, these key performance indicators will be included in the data collected for evaluation purposes. Additionally, all team members will be invited to complete a quantitative survey utilising validated instruments to assess their view of the team’s safety culture ^25^, team climate ^26^ and collective leadership ^27, 28^ at baseline and post-intervention.

### Step 4. Data analysis

A retroductive approach to data analysis will be adopted to analyse each case ^29^. This approach, advocated in realist research, uses both inductive and deductive logic to encourage researchers to think through the causal factors that may operate to produce certain observed programme outcomes ^29^. These causal factors may be hidden or implicit and therefore retroductive approaches require the researcher to use expertise and common sense to explore generative causation and to provide insight into factors influencing outcomes. Retroduction moves back and forth between indictive and deductive logic to interrogate both cases that are consistent and inconsistent with the IPT to enable confirmation, refinement as analysis progresses ^29^. Results will be discussed, and consensus reached, on refining the IPT during research team meetings.

### Step 5: Synthesis

The last step of the process will involve the completion of in-case analyses using the various data sources to triangulate findings within each case and compare the findings with the IPT. Next, a cross-comparison of each case to the CMOCs in the initial programme theory will be conducted to develop plausible hypotheses regarding how various contexts have triggered (or inhibited) particular mechanisms to lead to specific outcomes. It is anticipated that this process will inform the future development of a middle range theory that will be both relevant to all the cases studied and that may be generalised to other contexts where a collective leadership intervention is delivered. In realist evaluation, theory is never finalised, and this middle range would form a starting point a subsequent evaluation and theory refinement.

***Quality control.*** All qualitative data collected will be organised and managed using NVivo software ^30^. Analysis of interview transcripts and observation notes will be cross-checked by at least two researchers before agreement is reached on refining the programme theory and CMOCs. Finally, we will adhere to the RAMESES II best practice guidelines for the reporting of realist evaluations ^20^.

## Ethics

Favourable ethical opinion for the research has been obtained from the University College Dublin Research Ethics Committee (ref: HREC-LS-16-116397). This phase of the research began in December 2017 and will be completed by October 2019.

Informed consent will be sought from all participants in advance of their team’s participation in the intervention. We will confirm in participant information sheets that all data collected during the intervention will be confidential and that data will be aggregated so that individuals will not be identifiable from their responses or quotes. Unique personal identifiers will be employed so that the research team can link survey responses pre- and post-intervention.

## Discussion

This protocol elaborates on the approach and methods that will be adopted in developing a programme theory for the realist evaluation of a collective leadership intervention in healthcare settings ^31^. This paper describes a systematic and iterative approach to the development and testing of a programme theory to evaluate the impact of a collective leadership intervention. Realist evaluation is a method that enables the consideration of context and explores how an intervention may operate differently within different contexts to enable or inhibit certain mechanisms that lead to specific outcomes. It is the most appropriate approach to evaluation, exploring the implementation and effectiveness of complex interventions in complex, open systems ^16, 18^.

This work will significantly contribute to the emerging theory and developing evidence base for collective leadership in healthcare settings as it will address an identified gap in the literature by offering insight into how the intervention may operate, rather than just whether it is effective or not. This work will evaluate the impact of the intervention on the four teams and study sites and will inform the decision and approach on whether to further test the same intervention or a modified version of the intervention on a larger scale ^31^. The findings will also be used to refine the IPT and to inform the future development of a middle range theory (following further evaluation in a subsequent study exploring the impact of the intervention on a larger scale). The research will also help to inform future implementation strategies, given the insight that will be provided through this realist evaluation on contexts that may be more or less receptive to this type of intervention.

## Dissemination

We will disseminate out findings via peer-reviewed journals, targeted policy briefs to stakeholders and interest groups, present results at national and international conferences and circulate regular research updates via our research newsletter, social media and through updates on the dedicated research programme website. The results will also be fed back to participants: one report on progress near the mid-point of their involvement with the intervention and the second following completion of the realist evaluation post-intervention.

## Data availability

All data underlying the results are available as part of the article and no additional source data are required.
