# Supplementary material for: Understanding the impact of a collective leadership intervention on team working and safety culture in healthcare teams: a realist evaluation protocol
Source: HRB Open Res. 2020 Mar 26;2:5. Originally published 2019 Feb 6. [Version 2] doi: 10.12688/hrbopenres.12860.2 (PMC7140778; doi:10.12688/hrbopenres.12860.2)
Supplement: Peer review history

# Peer review: Reviewer response for version 2

**DOI:** 10.21956/hrbopenres.14098.r27268

**Published:** 2020-04-02

**Authors:** Sheena M. McHugh

**Affiliations:** 1 School of Public Health, University College Cork (UCC), Cork, Ireland

The authors have addressed my queries and comments. Only two minor points remaining:

- In the introduction, the authors clarify that *'this co-design process and the resulting intervention has been described in a previous paper and has already been evaluated'*, including a reference to the co-design publication. Has the evaluation been published? If so, you could include that reference here.
- There is a change of tense in the new text describing the systematic review and there are a couple of typos in the next text.

I confirm that I have read this submission and believe that I have an appropriate level of expertise to confirm that it is of an acceptable scientific standard.

# Peer review: Reviewer response for version 1

**DOI:** 10.21956/hrbopenres.13927.r26526

**Published:** 2019-04-24

**Authors:** Joanne Greenhalgh

**Affiliations:** 1 School of Sociology and Social Policy, University of Leeds, Leeds, UK

This is a protocol for a realist evaluation of a collective leadership intervention. The protocol is largely very clear and well described but there are some areas that require further elaboration:

1. While the aims and objectives have a realist flavor to them, the research questions seem to focus on questions of effectiveness. There seems to be some mismatch between the research questions (which focus on - does the intervention work and lead to the desired outcomes?) and the objectives (which focus on how does the intervention work). It would be helpful to have greater alignment between the two.
2. It is not clear exactly what the evaluand is – is it co-production and subsequent implementation of a collective leadership intervention or is JUST implementation of a collective leadership intervention? To help clarify this it would be helpful if the authors provided more detail about what is being done in the name of the intervention and how it is expected this will promote collective leadership and clarify whether those involved in the co-production of the intervention will also be the implementation sites (thus making the intervention co-production plus implementation of a collective leadership intervention – where drawing a line between the two is difficult) or whether the sites will be different (thus making it implementation of a collective leadership intervention)
3. Linked to the first point in (2) – it would be great to see an articulation of some candidate programme theories to be tested – this will help make sense of and justify the sampling and data collection choices.
4. The authors plan to conduct a realist synthesis to help formulate programme theories.  A full realist synthesis is a significant undertaking and goes further than simply identifying programme theories – it also involves theory testing and refining. It would be helpful if the authors further elaborated the nature of the synthesis and its relationship to the evaluation. The authors indicate that they plan to re-analyse papers included in a systematic review. The papers included in a systematic review often focus on effectiveness and are not always rich with programme theories. If the purpose of the realist synthesis is to elicit programme theories (rather than test and refine them) then they may need to look at different sorts of literature to identify these theories.
5. It would be great to see further elaboration of how data collection and analysis will support the process of theory testing throughout the study. The protocol seems to suggest that much of this will happen at the end, once data has all be collected.  However, theory testing and refinement is usually an ongoing process and occurs throughout data collection – often aided by the use of realist interviews based on the teacher learner cycle (eg see Manzano 2016 ^1^). 

I confirm that I have read this submission and believe that I have an appropriate level of expertise to confirm that it is of an acceptable scientific standard.

## References

[1] : The craft of interviewing in realist evaluation. *Evaluation*.2016;22(3) : 10.1177/1356389016638615 342-360 10.1177/1356389016638615

# Response

**Published:** 2020-02-25

**Authors:** Aoife De Brun

**Affiliations:** University College Dublin, Ireland

We would like to thank the reviewer for their detailed review and helpful comments, which we believe has strengthened the paper.

*“While the aims and objectives have a realist flavor to them, the research questions seem to focus on questions of effectiveness. There seems to be some mismatch between the research questions (which focus on - does the intervention work and lead to the desired outcomes?) and the objectives (which focus on how does the intervention work). It would be helpful to have greater alignment between the two.”*

 Thank you. We have revised the research questions to more clearly emphasise the realist focus and to ensure they are aligned to the objectives.

*“It is not clear exactly what the evaluand is – is it co-production and subsequent implementation of a collective leadership intervention or is JUST implementation of a collective leadership intervention? To help clarify this it would be helpful if the authors provided more detail about what is being done in the name of the intervention and how it is expected this will promote collective leadership and clarify whether those involved in the co-production of the intervention will also be the implementation sites (thus making the intervention co-production plus implementation of a collective leadership intervention – where drawing a line between the two is difficult) or whether the sites will be different (thus making it implementation of a collective leadership intervention)”*

 We have now clarified that the focus on this evaluation is on the implementation of the collective leadership intervention only. As suggested, we have included further detail regarding the intervention to make this explicit.

*“Linked to the first point in (2) – it would be great to see an articulation of some candidate programme theories to be tested – this will help make sense of and justify the sampling and data collection choices.”*

 We have now included two of the initial candidate theories underpinning the research programme (pages 9-10.

*“The authors plan to conduct a realist synthesis to help formulate programme theories.  A full realist synthesis is a significant undertaking and goes further than simply identifying programme theories – it also involves theory testing and refining. It would be helpful if the authors further elaborated the nature of the synthesis and its relationship to the evaluation. The authors indicate that they plan to re-analyse papers included in a systematic review. The papers included in a systematic review often focus on effectiveness and are not always rich with programme theories. If the purpose of the realist synthesis is to elicit programme theories (rather than test and refine them) then they may need to look at different sorts of literature to identify these theories.”*

 Thank you for this point and we agree that a realist synthesis is a very different undertaking as compared to a systematic review. However, our systematic review was conducted with the purpose of identifying approaches to developing collective leadership. Studies were eligible if they reported on the development, evaluation and/or implementation of training or interventions to foster collectivistic approaches to leadership. With this strong focus on development and implementation, as compared to a review that is focused on effectiveness only, we synthesised many papers that were rich in detail and provided a good starting point in the theory-building process. Given the known paucity of both peer-review and grey literature in this area, we have planned a series of additional steps to ensure the IPT can be tested and refined prior to the realist evaluation. We will interview members of teams who are currently adopting a collective leadership approach to their daily practice. This will support theory testing and refinement. Finally, the expert panel will also inform this process given their expertise in collective leadership, safety culture and team interventions. We have revised this section to more clearly explain this.

*“It would be great to see further elaboration of how data collection and analysis will support the process of theory testing throughout the study. The protocol seems to suggest that much of this will happen at the end, once data has all be collected.  However, theory testing and refinement is usually an ongoing process and occurs throughout data collection – often aided by the use of realist interviews based on the teacher learner cycle (eg see Manzano 2016* *).”*

 Thank you for this. Data collection will be staggered through the realist evaluation cycle, as is typical in realist approach. Data will be collected from teams participating in the realist evaluation over the period of months. This will enable the research team to ensure theory testing and refinement throughout the evaluation process through using Manzano’s theory-based testing approach to interviewing. This is now clarified in the Methods section (page 12).

# Peer review: Reviewer response for version 1

**DOI:** 10.21956/hrbopenres.13927.r26504

**Published:** 2019-03-07

**Authors:** Sheena M. McHugh

**Affiliations:** 1 School of Public Health, University College Cork (UCC), Cork, Ireland

This manuscript describes the protocol for a realist evaluation of a collective leadership intervention in healthcare. The rationale for this approach is clearly outlined and the authors describe in detail how the principles of realist evaluation will be operationalised. 

 In relation to the objectives, there appears to be some overlap between objectives 1 and 4. Similarly, objective 3 (assessing outcomes), overlaps with objective 2 (to describe how the intervention is working). This could be addressed by reordering the objectives to show the development of knowledge from description of outcomes to identifying contexts and mechanisms related to these outcomes. 

 The manuscript would benefit from greater clarity on the implementation of the collective leadership intervention and sampling of teams. Has the intervention been implemented across all teams in the hospital group and 4 teams who received the intervention were selected for this evaluation? When was the intervention implemented? This has implications for the assessment of its impact. 

 Related to this, over what time period is the evaluation being conducted?

 In terms of the selection of teams, it states in the 'context of research' section that four different team types were selected based on diversity and in line with priorities. From what pool were these teams selected (4 out of how many teams)? How was the 'type' defined/categorised? 

 In the section outlining interviews with individuals on teams working collectively, have these teams received the intervention? If these are the same teams in the case study, where one of the quantitatively assessed outcomes is collective leadership, the authors need to address the circularity in this (selection on basis of perceived collective working and outcome is collective leadership). This is particularly important to clarify given the first research question. 

 The authors refer to a particular taxonomy to define a team (ref 21). Is this a taxonomy for defining collective working or simply for defining a team? If it defines a team, how was collective working aspect assessed? 

 Are the team members interviewed during the development of the IPT, the same teams and team members involved in the case studies to refine the theory? This is not clear in the manuscript or the abstract. 

 In relation to the realist synthesis, I wonder whether the papers included in a systematic review of effectiveness will provide sufficient information to develop an IPT. For example, there may be process evaluations or other literature attached to the interventions which outline contextual factors that influenced effectiveness.

 Minor comments

 In the abstract, the authors state that the realist evaluation approach 'enables' the use of mixed-methods to explore the research question. However, they also correctly state multiple times in the text that this approach is method-neutral thus I would suggest rephrasing the abstract. 

 In objective 2, the inclusion of (mechanism) after 'specific implementation contexts' is confusing given both are distinct elements of CMO configurations.

I confirm that I have read this submission and believe that I have an appropriate level of expertise to confirm that it is of an acceptable scientific standard, however I have significant reservations, as outlined above.

# Response

**Published:** 2020-02-25

**Authors:** Aoife De Brun

**Affiliations:** University College Dublin, Ireland

We would again like to thank the reviewer for their detailed review and helpful comments, which we believe has strengthened the paper.

*“In relation to the objectives, there appears to be some overlap between objectives 1 and 4. Similarly, objective 3 (assessing outcomes), overlaps with objective 2 (to describe how the intervention is working). This could be addressed by reordering the objectives to show the development of knowledge from description of outcomes to identifying contexts and mechanisms related to these outcomes.”*

 Thank you. We have amended the objectives for clarity and to remove repetition (page 8)

*“The manuscript would benefit from greater clarity on the implementation of the collective leadership intervention and sampling of teams. Has the intervention been implemented across all teams in the hospital group and 4 teams who received the intervention were selected for this evaluation? When was the intervention implemented? This has implications for the assessment of its impact. Related to this, over what time period is the evaluation being conducted?”*

*“In terms of the selection of teams, it states in the 'context of research' section that four different team types were selected based on diversity and in line with priorities. From what pool were these teams selected (4 out of how many teams)? How was the 'type' defined/categorised?”*

 Thank you for raising these relevant points. We have revised the paper to address these issues and clarify the process around the selection of teams and implementation (page 11). Whilst we have not categorised ‘type’ of team. We have collaborated with the hospital group management team identifying a range of healthcare teams that will provide different contexts and ways of working to enable a rich testing of our programme theory.

*“In the section outlining interviews with individuals on teams working collectively, have these teams received the intervention? If these are the same teams in the case study, where one of the quantitatively assessed outcomes is collective leadership, the authors need to address the circularity in this (selection on basis of perceived collective working and outcome is collective leadership). This is particularly important to clarify given the first research question.”*

*“Are the team members interviewed during the development of the IPT, the same teams and team members involved in the case studies to refine the theory? This is not clear in the manuscript or the abstract.”*

 No. These teams are separate to those who took part in the intervention and had no role in the co-design or implementation of the intervention of study. These teams were identified by leaders in the healthcare systems as already operating collectively. We have now made this explicit in the text (page 10) and in the abstract.

*“The authors refer to a particular taxonomy to define a team (ref 21). Is this a taxonomy for defining collective working or simply for defining a team? If it defines a team, how was collective working aspect assessed?”*

 This taxonomy is relevant to how we have defined a team for the purposes of this work. The collective working aspects was assessed using criteria that had emerged from our systematic review.  This has been clarified on page 12.

*“In relation to the realist synthesis, I wonder whether the papers included in a systematic review of effectiveness will provide sufficient information to develop an IPT. For example, there may be process evaluations or other literature attached to the interventions which outline contextual factors that influenced effectiveness.”*

 Thank you. Yes, this point was also raised by Reviewer 1 and we have responded above. We are aware there is a lack of research in this area (peer-review and grey literature) and therefore have outlined this multi-phased approach which includes interviewing additional teams working collectively and consultation with an expert panel (external subject-matter experts and programme designers) to allow for additional input. We are confident this will ensure sufficient information and insights to facilitate the development of an informed IPT.

*“In the abstract, the authors state that the realist evaluation approach 'enables' the use of mixed-methods to explore the research question. However, they also correctly state multiple times in the text that this approach is method-neutral thus I would suggest rephrasing the abstract.”*

 Thank you. This has been amended.
